# Effectiveness and safety of pembrolizumab for the treatment of Japanese patients with microsatellite instability-high tumors excluding colorectal cancer: a post-marketing surveillance

**DOI:** 10.1093/jjco/hyaf064

**Published:** 2025-05-18

**Authors:** Daisuke Aoki, Mai Yamauchi, Makiko Izawa, Yuichiro Ito, Masahiro Hamada, Masahiko Ozaki, Shinichiroh Maekawa, Kei Muro

**Affiliations:** International University of Health and Welfare Graduate School, W 4-1-26 Akasaka, Minato-ku, Tokyo 107-8402, Japan; Akasaka Sanno Medical Center, W 4-1-26 Akasaka, Minato-ku, Tokyo 107-8402, Japan; Medical Affairs Oncology, MSD K.K., Kitanomaru Square, 1-13-12 Kudan-kita, Chiyoda-ku, Tokyo 102-8667, Japan; Medical Affairs Oncology, MSD K.K., Kitanomaru Square, 1-13-12 Kudan-kita, Chiyoda-ku, Tokyo 102-8667, Japan; Medical Affairs Oncology, MSD K.K., Kitanomaru Square, 1-13-12 Kudan-kita, Chiyoda-ku, Tokyo 102-8667, Japan; Pharmacovigilance, MSD K.K., Kitanomaru Square, 1-13-12 Kudan-kita, Chiyoda-ku, Tokyo 102-8667, Japan; Pharmacovigilance, MSD K.K., Kitanomaru Square, 1-13-12 Kudan-kita, Chiyoda-ku, Tokyo 102-8667, Japan; Pharmacovigilance, MSD K.K., Kitanomaru Square, 1-13-12 Kudan-kita, Chiyoda-ku, Tokyo 102-8667, Japan; Department of Clinical Oncology, Aichi Cancer Center Hospital, 1-1 Kanokoden, Chikusa-ku, Nagoya 464-8681, Japan

**Keywords:** cancer, endometrial cancer, Japan, microsatellite instability, pembrolizumab

## Abstract

**Background:**

We aimed to assess the real-world effectiveness and safety of pembrolizumab monotherapy in Japanese patients with high-frequency microsatellite instability (MSI-H) solid tumors except colorectal cancer.

**Methods:**

This multicenter, observational, post-marketing surveillance had a 12-month observation period. We included all patients with locally advanced or metastatic MSI-H solid tumors, except colorectal cancer, in whom standard treatment was difficult or who had shown tumor progression after conventional chemotherapies and had started treatment with pembrolizumab by 31 December 2019.

**Results:**

In total, 403 patients were enrolled, and 396 and 376 patients were included in the safety and effectiveness analysis sets, respectively. The numbers of patients and frequencies of tumor types occurring in ≥20 cases were: endometrial, 162/403 (40.2%); gastric, 61/403 (15.1%); biliary tract, 42/403 (10.4%); pancreatic, 29/403 (7.2%); and ovarian, 20/403 (5.0%). The objective response rate was 50.3% (189/376) and the disease control rate was 71.5% (269/376). The 12-month progression-free survival (PFS) rate was 42.1% and the median PFS was 8.8 months (95% confidence interval, 6.4–11.5). The 12-month overall survival (OS) rate was 75.1%, and median OS was not reached. Treatment-related adverse events (AEs) of special interest of any grade occurred in 128/396 (32.3%) patients, and those of Grade ≥ 3, in 54/396 (13.6%) patients. One patient with esophageal cancer experienced a Grade 5 AE. No new safety signals were observed.

**Conclusions:**

This study confirmed the real-world effectiveness and safety of pembrolizumab monotherapy in patients with MSI-H solid tumors except colorectal cancer in Japan.

## Teaser

This post-marketing surveillance confirmed the real-world effectiveness and safety of pembrolizumab monotherapy in patients with high-frequency microsatellite instability solid tumors except colorectal cancer in Japan.

## Graphical Abstract

**Graphical Abstract:**
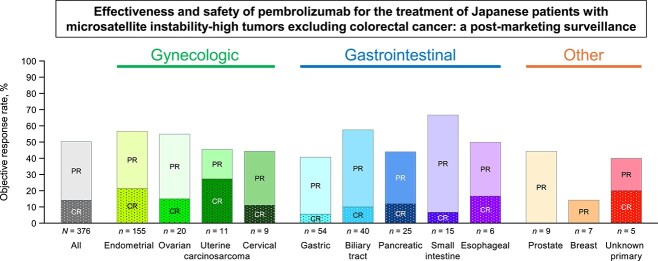

## Introduction

Microsatellites are short, tandem DNA sequences of mononucleotide, dinucleotide, or higher-order nucleotide repeats that are scattered throughout the human genome [1]. Microsatellite regions are prone to DNA replication errors because of DNA polymerase slippage, leading to mismatched strands. Although DNA polymerase attempts to correct DNA replication errors, some errors escape repair and are corrected by mismatch repair (MMR) proteins. This leads to the development of high-frequency microsatellite instability (MSI-H) or MMR deficiency (dMMR) tumors [2]. The tumor types with the highest rates of MSI-H and dMMR prevalence are colorectal, gastric, and endometrial carcinoma [3,4]. MSI status is confounded by disease stage [5]. In Japan, the following three methods have been approved as companion diagnostics to measure the MSI/MMR status in tumors: polymerase chain reaction (PCR) and next-generation sequencing to determine MSI status and immunohistochemistry to determine MMR status. A study using PCR-based MSI testing on 26 237 samples of various cancer types in Japan reported an overall MSI-H frequency of 3.72%, and MSI-H was detected in 30 cancer types [6]. The most frequent cancer types were endometrial cancer (16.85%), small intestine cancer (8.63%), gastric cancer (6.74%), duodenal cancer (5.60%), and colorectal cancer (3.78%) [6].

KEYNOTE-016 was a phase 2 study that evaluated the clinical activity of pembrolizumab, an anti-programmed cell death protein 1 (PD-1) monoclonal antibody, in patients with progressive metastatic carcinoma with or without dMMR [7,8]. The results showed that dMMR tumors were more responsive to PD-1 blockade with pembrolizumab than MMR-proficient tumors. In May 2017, pembrolizumab was first approved in the United States for the treatment of patients with unresectable or metastatic MSI-H/dMMR solid tumors that are not responsive to prior therapy and for which no other treatment options are available based on data from the KEYNOTE-164 study (which included patients with colorectal tumors) [9] and the KEYNOTE-158 study (which included patients with non-colorectal tumors) [10]. In Japan, the application for approval was submitted using data from the KEYNOTE-164 study (Cohort A) [9] and the KEYNOTE-158 study, including the Japanese population as reference data [10], and in December 2018, pembrolizumab received conditional early approval for the additional indication of advanced or recurrent MSI-H solid tumors that have progressed following prior treatment and for patients who have no satisfactory alternative treatment options. Updated data from the KEYNOTE-164 (Cohort B) study showed the clinical benefit of pembrolizumab in patients with unresectable or metastatic MSI-H/dMMR colorectal tumors treated with ≥1 prior line of therapy [9], and KEYNOTE-177 confirmed the clinical benefit of pembrolizumab as first-line therapy for patients with metastatic MSI-H/dMMR colorectal cancer [11]. Consequently, pembrolizumab was approved for unresectable or metastatic MSI-H/dMMR colorectal cancer in the United States in June 2020 based on the results of KEYNOTE-177 and for unresectable or metastatic MSI-H colorectal cancer in Japan in August 2021 based on the results of both KEYNOTE-177 and KEYNOTE-164 (Cohort B). In an updated analysis of Cohort K of the KEYNOTE-158 study, which included 351 patients with previously treated advanced MSI-H/dMMR non-colorectal tumors (median time from first dose to data cutoff: 37.5 months), the overall survival (OS) rate was 39.1% at both 3 and 4 years (median OS, 20.1 months; 95% confidence interval [CI], 14.1–27.1 months) [12]. Therefore, PD-1 inhibitors are strongly recommended for the treatment of patients with MSI-H/dMMR solid tumors based on the evidence from clinical trials of pembrolizumab [13,14].

Of note, the number of Japanese patients enrolled in previous clinical trials has been limited, and the real-world effectiveness and safety of pembrolizumab in Japanese patients with advanced or relapsed MSI-H solid tumors after chemotherapy are currently unknown. In KEYNOTE-158, only seven Japanese patients with non-colorectal cancer were included, and the number of patients with each tumor type was limited.

The objective of the present study was to assess the real-world effectiveness and safety of pembrolizumab monotherapy in Japanese patients with MSI-H solid tumors except for colorectal cancer. Patients with colorectal cancer were not included in this study because six Japanese patients with colorectal cancer had already been included in the KEYNOTE-164 trial, and data collection on colorectal cancer cases was not requested by the Pharmaceuticals and Medical Devices Agency in Japan.

## Patients and methods

### Study design and patients

This was a multicenter, observational, post-marketing surveillance, including 251 participating medical institutions. All patients with locally advanced or metastatic MSI-H solid tumors, except colon and rectal cancers, were included if they had no history of previous chemotherapy due to a lack of alternative treatment options or if they had shown tumor progression after conventional chemotherapies. Additionally, patients must have started treatment with pembrolizumab by 31 December 2019. Pembrolizumab was administered at a dose of 200 mg by intravenous infusion over 30 min every 3 weeks or 400 mg by intravenous infusion over 30 min every 6 weeks.

Patients were registered from 21 December 2018 to 20 December 2021 using a central registration method. The observation period was 12 months from the start of treatment with pembrolizumab. The survey forms were collected after 6 and 12 months of treatment. When patients discontinued treatment before 12 months, the observation period was until the date of discontinuation. When patients developed adverse events of special interest (AEOSI), the observation period was until 30 days after the end of treatment. For cases in which follow-up was difficult due to hospital change before the end of the observation period, the observation period was until the last visit date. The data cutoff was set to 20 June 2022.

The study was conducted in accordance with the Declaration of Helsinki and adhered to Good Post-Marketing Study Practice (GPSP) authorized by the Ministry of Health, Labour and Welfare of Japan. The GPSP exempts the requirement of informed consent from patients for post-marketing surveillance, considering that the surveillance only involves confirming treatment results observed in daily clinical practice and does not involve any additional treatments or examinations beyond standard care.

### Effectiveness assessments

Tumor response was evaluated by each attending physician, according to the Response Evaluation Criteria in Solid Tumors (RECIST) version 1.1. The objective response rate (ORR) was evaluated and defined as the proportion of patients with a complete response (CR) or partial response (PR). The proportions of patients with progressive disease (PD) or stable disease (SD) were also recorded. The disease control rate (DCR) was evaluated and defined as the proportion of patients with CR, PR, or SD. The time to response was defined as the time from the start of treatment to the date of the first documented CR or PR. Duration of response was defined as the time from the first documented CR or PR to the date of the first documented PD or death, whichever occurred first. Progression-free survival (PFS) was defined as the time from the start of treatment to the date of the first documented PD or death. OS was defined as the time from the start of treatment to the date of death.

### Safety assessments

Adverse events (AEs) were coded using the Medical Dictionary for Regulatory Activities/Japanese version 25.0 and graded using the Common Terminology Criteria for AEs version 5.0 from the Japanese Clinical Oncology Group. Only AEOSI that were specified for safety monitoring were collected.

### Statistical methods

The planned sample size was at least 30 patients (at least 20 patients for the effectiveness analysis). Based on data from KEYNOTE-158, the ORR in 94 patients with MSI-H/dMMR non-colorectal cancer was 37.2%. Assuming an expected ORR of 5% for the null hypothesis, the number of cases needed to reject the null hypothesis at alpha = 2.5% (one-tailed test) securing a 95% detection power was approximately 20 cases. Considering the possibility that approximately 10 patients would be excluded from the analysis target population, the target sample size was set at 30 patients.

The analysis populations for the present study were the safety analysis set and the effectiveness analysis set. Frequencies and percentages were calculated for categorical variables, and summary statistics were calculated for continuous data. PFS and OS were estimated using Kaplan–Meier analysis. Median PFS and OS and the corresponding 95% CIs were calculated. The safety analysis set (which represented the largest evaluable population) was used to include cases with data on death or disease progression (17 out of 20 cases had data on death or disease progression but did not have RECIST evaluation data). However, cases that could not be evaluated due to missing data were excluded from each analysis. All statistical analyses were performed using SAS software version 9.4 (SAS Institute Inc., Cary, NC, USA).

## Results

### Patients

**Figure 1:**
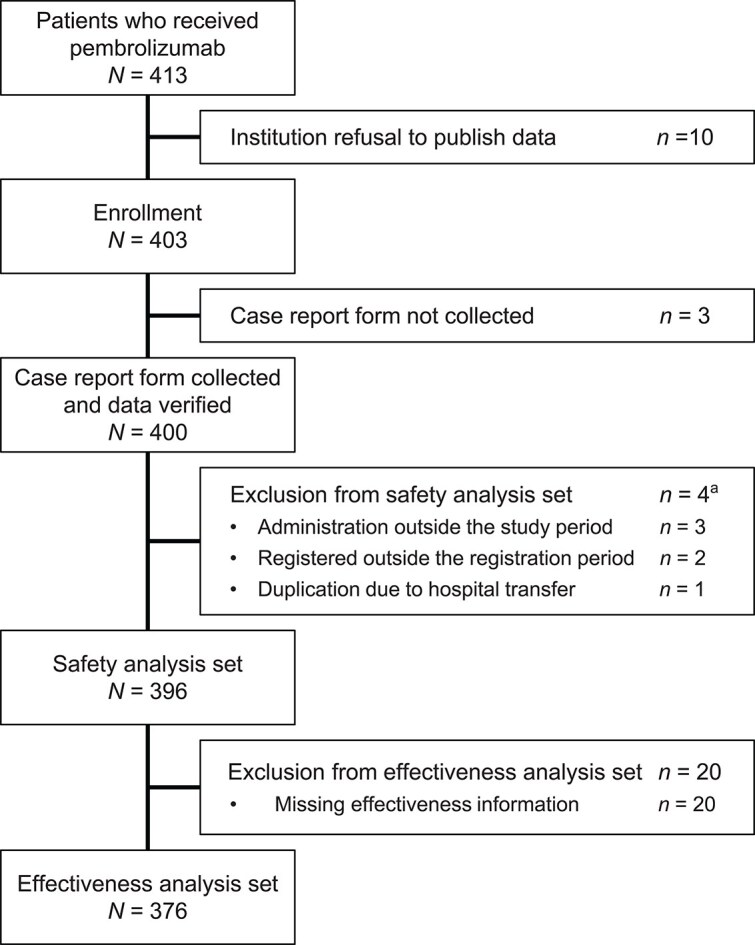
Patient disposition. ^a^Patients may have been excluded for more than one reason.

Among 413 patients who received pembrolizumab, 403 were enrolled because 10 patients were treated at institutions that did not consent to publication of the data. Case report forms were obtained from 400 patients (Fig. 1). In total, 396 patients were included in the safety analysis set and 376 in the effectiveness analysis set.

The most frequent tumor type among all patients enrolled in the present study was endometrial in 162/403 (40.2%) patients, followed by gastric in 61/403 (15.1%), biliary tract in 42/403 (10.4%), pancreatic in 29/403 (7.2%), and ovarian in 20/403 (5.0%) patients (Supplementary Table S1). The baseline demographic and clinical characteristics of patients in the safety analysis set (*N* = 396) and by tumor type (for tumors that occurred in ≥20 cases) are summarized in Supplementary Table S2. Overall, most patients were female (71.7%) and the median age was 63.0 years. Most patients had an Eastern Cooperative Oncology Group performance status (ECOG PS) of 0 (52.0%) or 1 (33.6%), 44.4% had received pre-operative/post-operative chemotherapy, and 68.2% of patients had received 1–2 prior lines of therapy. The median ages were 58.0, 72.0, 69.0, 69.0, and 49.0 years among the patients with endometrial, gastric, biliary tract, pancreatic, and ovarian cancer, respectively. The proportions of patients with an ECOG PS of ≤1 and ≥2 were 84.4% and 15.6% among those with endometrial cancer, 90.2% and 9.8% among those with gastric cancer, 90.5% and 9.5% among those with biliary tract cancer, 82.1% and 17.9% among those with pancreatic cancer, and 90.0% and 10.0% among those with ovarian cancer, respectively. Among the 160 patients with endometrial cancer, 54 (33.8%) and 66 (41.3%) had undergone one and two prior line(s) of chemotherapy (including pre-operative/post-operative chemotherapy), respectively. Similarly, more than 50% of patients with cancer types other than endometrial had undergone one or two prior line(s) of chemotherapy, but pre-operative/post-operative chemotherapy was not included as a prior line of therapy when analyzing the data for the other cancer types. The baseline characteristics of patients in the safety analysis set were similar to those in the effectiveness analysis set (*N* = 376) (Supplementary Table S3).

MSI testing results are shown in Supplementary Table S4. Most patients (90.4%) underwent PCR testing. The sample location was the primary site in 83.6% of cases. Among the 160 patients with endometrial cancer, surgical resection samples were used for testing in 130 (81.3%) patients. Among the 61 patients with gastric cancer, biopsy samples were used for testing in 36 (59.0%) patients, and surgical resection samples were used for testing in 25 (41.0%) patients.

### Effectiveness

#### Tumor response

The results related to tumor response in all patients and by tumor type are summarized in Table 1. Among the 376 patients in the effectiveness analysis set, the tumor response was CR in 53 patients (14.1%), PR in 136 (36.2%), SD in 80 (21.3%), and PD in 107 (28.5%). The ORR was 50.3% (189/376) and the DCR was 71.5% (269/376).

When considering patients with tumors that occurred in ≥20 cases, the percentage of patients who achieved CR was the highest at 21.3% in those with endometrial cancer. The percentage of patients who achieved PR was the highest at 47.5% in patients with biliary tract cancer, and for SD was the highest at 25.0% in patients with ovarian cancer.

The median duration of treatment was 29.4 weeks in all patients and was the longest at 49.4 weeks in patients with ovarian cancer. The median duration of response was 10.0 months in patients with biliary tract cancer, and was not reached for any other cancer type.

The results of tumor response by tumor type (for tumors that occurred in ≥5 cases in the overall enrolled population [*N* = 403]) including small intestine cancer (*n* = 16, 4.0%), uterine carcinosarcoma (*n* = 11, 2.7%), cervical cancer (*n* = 10, 2.5%), prostate cancer (*n* = 9, 2.2%), breast cancer (*n* = 7, 1.7%), unknown primary cancer (*n* = 7, 1.7%), and esophageal cancer (*n* = 6, 1.5%) are summarized in Table 1. The ORR was the highest at 66.7% (10/15) in patients with small intestine cancer.

**Table 1:** Summary of baseline demographic and clinical characteristics, response outcomes, and AEOSI by tumor type (for tumors that occurred in ≥5 cases).

**Cancer type**	**All**	**Endometrial**	**Gastric**	**Biliary tract**	**Pancreatic**	**Ovarian**	**Small intestine**	**Uterine carcinosarcoma**	**Cervical**	**Prostate**	**Breast**	**Unknown primary**	**Esophageal**
All enrolled patients, *N* (%)	403	162 (40.2)	61 (15.1)	42 (10.4)	29 (7.2)	20 (5.0)	16 (4.0)	11 (2.7)	10 (2.5)	9 (2.2)	7 (1.7)	7 (1.7)	6 (1.5)
Safety analysis set, *N*	396	160	61	42	28	20	16	11	9	9	7	5	6
Sex, female, *n* (%)	284 (71.7)	160 (100.0)	26 (42.6)	19 (45.2)	12 (42.9)	20 (100.0)	8 (50.0)	11 (100.0)	9 (100.0)	0	7 (100.0)	3 (60.0)	0
Age, median (range), years	63 (34–96)	58 (38–81)	72 (36–96)	69 (42–81)	69 (39–84)	49 (34–74)	62 (40–80)	56 (45–66)	47 (40–71)	72 (61–80)	57 (43–75)	52 (38–74)	77 (63–78)
ECOG PS 0, 1, *n* (%)	339 (85.6)	135 (84.4)	55 (90.2)	38 (90.5)	23 (82.1)	18 (90.0)	13 (81.3)	9 (81.8)	9 (100.0)	8 (88.9)	6 (85.7)	3 (60.0)	5 (83.3)
ECOG PS ≥2, *n* (%)	57 (14.4)	25 (15.6)	6 (9.8)	4 (9.5)	5 (17.9)	2 (10.0)	3 (18.8)	2 (18.2)	0	1 (11.1)	1 (14.3)	2 (40.0)	1 (16.7)
Distant metastasis, *n* (%)	321 (81.1)	124 (77.5)	56 (91.8)	34 (81.0)	22 (78.6)	16 (80.0)	12 (75.0)	9 (81.8)	6 (66.7)	8 (88.9)	7 (100.0)	5 (100.0)	3 (50.0)
Prior pre-operative/post-operative chemotherapy, *n* (%)	176 (44.4)	103 (64.4)	17 (27.9)	9 (21.4)	8 (28.6)	16 (80.0)	4 (25.0)	5 (45.5)	4 (44.4)	2 (22.2)	3 (42.9)	0	0
Prior lines of chemotherapy, *n* (%)													
0	5 (1.3)	2 (1.3)^a^	0	0	1 (3.6)	0	1 (6.3)	0	0	0	0	0	0
1	154 (38.9)	54 (33.8)^a^	23 (37.7)	15 (35.7)	10 (35.7)	5 (25.0)	8 (50.0)	5 (45.5)	3 (33.3)	2 (22.2)	0	3 (60.0)	1 (16.7)
2	116 (29.3)	66 (41.3)^a^	19 (31.1)	20 (47.6)	11 (39.3)	7 (35.0)	4 (25.0)	2 (18.2)	4 (44.4)	0	0	2 (40.0)	4 (66.7)
3	38 (9.6)	22 (13.8)^a^	6 (9.8)	3 (7.1)	3 (10.7)	2 (10.0)	1 (6.3)	1 (9.1)	1 (11.1)	1 (11.1)	2 (28.6)	0	1 (16.7)
≥4	40 (10.1)	14 (8.8)^a^	8 (13.1)	2 (4.8)	3 (10.7)	3 (15.0)	1 (6.3)	1 (9.1)	1 (11.1)	6 (66.7)	5 (71.4)	0	0
Unknown	5 (1.3)	2 (1.3)^a^	1 (1.6)	1 (2.4)	0	0	1 (6.3)	0	0	0	0	0	0
TR AEOSI (any grade), % (*n*/*N*)	32.3 (128/396)	34.4 (55/160)	27.9 (17/61)	40.5 (17/42)	21.4 (6/28)	35.0 (7/20)	25.0 (4/16)	45.5 (5/11)	44.4 (4/9)	22.2 (2/9)	28.6 (2/7)	60.0 (3/5)	33.3 (2/6)
TR AEOSI (Grade ≥3), % (*n*/*N*)	13.6 (54/396)	13.8 (22/160)	8.2 (5/61)	21.4 (9/42)	3.6 (1/28)	10.0 (2/20)	12.5 (2/16)	36.4 (4/11)	22.2 (2/9)	22.2 (2/9)	0.0 (0/7)	0.0 (0/5)	16.7 (1/6)
Time on treatment, median, weeks	25.7	38.5	12.1	38.1	17.9	49.4	49.9	22.1	22.0	30.7	7.1	25.1	18.0
PFS, median (95% CI), months	8.8 (6.4–11.5)	NR (8.0–NR)	4.0 (2.4–7.1)	11.5 (3.8–NR)	5.7 (2.3–NR)	9.0 (5.6–NR)	12.2 (10.5–NR)	8.8 (1.8–NR)	6.7 (1.3–NR)	9.0 (0.1–NR)	1.5 (0.7–2.1)	NR (2.4–NR)	4.4 (0.7–NR)
6-month PFS rate, %	56.0	63.7	40.6	60.6	48.1	75.0	87.5	54.5	53.3	55.6	14.3	80.0	44.4
12-month PFS rate, %	42.1	53.8	29.6	30.2	37.0	47.7	70.7	43.6	40.0	18.5	14.3	53.3	22.2
OS, median (95% CI), months	NR (NR–NR)	NR (NR–NR)	NR (NR–NR)	NR (NR–NR)	NR (3.7–NR)	NR (NR–NR)	NR (12.2–NR)	NR (2.4–NR)	NR (3.6–NR)	NR (2.0–NR)	NR (1.1–NR)	NR (10.4–NR)	11.7 (1.2–NR)
6-month OS rate, %	79.6	85.4	72.9	77.7	70.2	94.4	87.5	71.6	87.5	77.8	53.6	100.0	83.3
12-month OS rate, %	75.1	81.9	69.4	77.7	63.2	87.2	87.5	71.6	70.0	62.2	53.6	66.7	41.7
Effectiveness analysis set, *N*	376	155	54	40	25	20	15	11	9	9	7	5	6
OR, % (*n*/*N*)^b^	50.3 (189/376)	56.8 (88/155)	40.7 (22/54)	57.5 (23/40)	44.0 (11/25)	55.0 (11/20)	66.7 (10/15)	45.5 (5/11)	44.4 (4/9)	44.4 (4/9)	14.3 (1/7)	40.0 (2/5)	50.0 (3/6)
DC, % (*n*/*N*)	71.5 (269/376)	73.5 (114/155)	61.1 (33/54)	80.0 (32/40)	68.0 (17/25)	80.0 (16/20)	93.3 (14/15)	63.6 (7/11)	66.7 (6/9)	66.7 (6/9)	28.6 (2/7)	100.0 (5/5)	66.7 (4/6)
CR, % (*n*/*N*)	14.1 (53/376)	21.3 (33/155)	5.6 (3/54)	10.0 (4/40)	12.0 (3/25)	15.0 (3/20)	6.7 (1/15)	27.3 (3/11)	11.1 (1/9)	0.0 (0/9)	0.0 (0/7)	20.0 (1/5)	16.7 (1/6)
PR, % (*n*/*N*)	36.2 (136/376)	35.5 (55/155)	35.2 (19/54)	47.5 (19/40)	32.0 (8/25)	40.0 (8/20)	60.0 (9/15)	18.2 (2/11)	33.3 (3/9)	44.4 (4/9)	14.3 (1/7)	20.0 (1/5)	33.3 (2/6)
SD, % (*n*/*N*)	21.3 (80/376)	16.8 (26/155)	20.4 (11/54)	22.5 (9/40)	24.0 (6/25)	25.0 (5/20)	26.7 (4/15)	18.2 (2/11)	22.2 (2/9)	22.2 (2/9)	14.3 (1/7)	60.0 (3/5)	16.7 (1/6)
PD, % (*n*/*N*)	28.5 (107/376)	26.5 (41/155)	38.9 (21/54)	20.0 (8/40)	32.0 (8/25)	20.0 (4/20)	6.7 (1/15)	36.4 (4/11)	33.3 (3/9)	33.3 (3/9)	71.4 (5/7)	0.0 (0/5)	33.3 (2/6)
Time on treatment, median, weeks	29.4	40.1	15.6	43.1	18.1	49.4	50.1	22.1	22.0	30.7	7.1	25.1	18.0
DOR, median (range), months	NR (0.6–10.0)	NR (0.8–5.9)	NR (1.4–9.3)	10.0 (0.6–10.0)	NR (2.6–4.9)	NR (3.4–6.1)	NR (7.0–9.1)	NR (5.3–5.3)	NR (NR–NR)	8.1 (6.9–9.2)	NR (NR–NR)	NR (NR–NR)	NR (5.3–5.3)
TTR, median (range), months	4.5 (0.3–10.0)	4.5 (0.8–10.0)	4.7 (0.3–6.4)	2.7 (1.4–8.4)	5.3 (1.4–6.0)	4.4 (1.2–6.0)	3.3 (1.6–4.4)	4.4 (1.8–4.4)	NR (3.3–4.0)	4.7 (1.8–4.7)	NR (1.9–1.9)	NR (0.6–2.8)	4.3 (1.9–5.2)
DOR ≥6 months, *n* (%)^c^	121 (64.0)	53 (60.2)	17 (77.3)	17 (73.9)	6 (54.5)	8 (72.7)	9 (90.0)	2 (40.0)	2 (50.0)	3 (75.0)	1 (100.0)	2 (100.0)	1 (33.3)

^a^For patients with endometrial cancer, pre-operative/post-operative chemotherapy was included as prior lines of therapy. Other cancers did not include pre- or post-operative chemotherapy.

^b^Effectiveness evaluation (CR, PR, SD, and PD) was based on the best effectiveness evaluation per case.

^c^Percentages are based on the number of patients who achieved CR or PR.

PFS and OS were calculated in the safety analysis set.

Abbreviations: AEOSI, adverse events of special interest. CI, confidence interval. CR, complete response. DC, disease control. DOR, duration of response. ECOG PS, Eastern Cooperative Oncology Group performance status. NR, not reached. OR, objective response. OS, overall survival. PD, progressive disease. PFS, progression-free survival. PR, partial response. SD, stable disease. TR, treatment-related. TTR, time to response.

Table 2 shows the results of tumor response in patients with endometrial cancer according to the number of prior lines of therapy (including pre-operative/post-operative chemotherapy). Among the 155 patients with endometrial cancer, the tumor response was CR in 33 patients (21.3%), PR in 55 (35.5%), SD in 26 (16.8%), and PD in 41 (26.5%). The ORR was 56.8% (88/155) and the DCR was 73.5% (114/155). The tumor response was similar regardless of the number of prior lines of therapy. Among 152 patients with endometrial cancer, the median duration from prior chemotherapy to the start of treatment was 3 months in the effectiveness analysis set, excluding two patients with no prior platinum chemotherapy and one patient with unknown prior chemotherapy, including pre-operative/post-operative chemotherapy (Supplementary Table S5). Among the 152 patients with endometrial cancer who received pembrolizumab and were assessed by platinum-free interval (i.e. the time between last platinum chemotherapy and recurrence) [15–17], the majority had a platinum-free interval of <6 months (*n* = 100), followed by 6 to <12 months (*n* = 29), and ≥12 months (*n* = 21) (two patients did not receive prior platinum therapy). The response outcome of endometrial cancer by platinum-free interval is shown in Supplementary Table S6. The ORR in patients with a platinum-free interval <6 months was 57.0%; 6 to <12 months, 58.6%; and ≥12 months, 47.6%. The DCR in patients with a platinum-free interval <6 months was 70.0%; 6 to <12 months, 79.3%; and ≥12 months, 81.0%.

**Table 2:** Response outcome of endometrial cancer by prior lines of therapy (effectiveness analysis set).

	**Endometrial**		**One line of prior therapy** ^a^		**Two or more lines of prior therapy** ^a^	
**Best overall response**	***N* = 155**		***n* = 53**		***n* = 98**	
OR	88	(56.8)	30	(56.6)	55	(56.1)
DC	114	(73.5)	37	(69.8)	74	(75.5)
CR	33	(21.3)	10	(18.9)	21	(21.4)
PR	55	(35.5)	20	(37.7)	34	(34.7)
SD	26	(16.8)	7	(13.2)	19	(19.4)
PD	41	(26.5)	16	(30.2)	24	(24.5)
Time to response, months	4.5	(0.8–10.0)	4.3	(0.8–9.7)	4.7	(1.2–10.0)
Duration of response, months	NR	(0.8–5.9)	NR	(4.7–5.6)	NR	(0.8–5.9)

^a^Number of prior lines of therapy include pre-operative/post-operative chemotherapy.

Data are *n* (%) or median (range).

Abbreviations: CR, complete response. DC, disease control. NR, not reached. OR, objective response. PD, progressive disease. PR, partial response. SD, stable disease.

#### Survival

**Figure 2:**
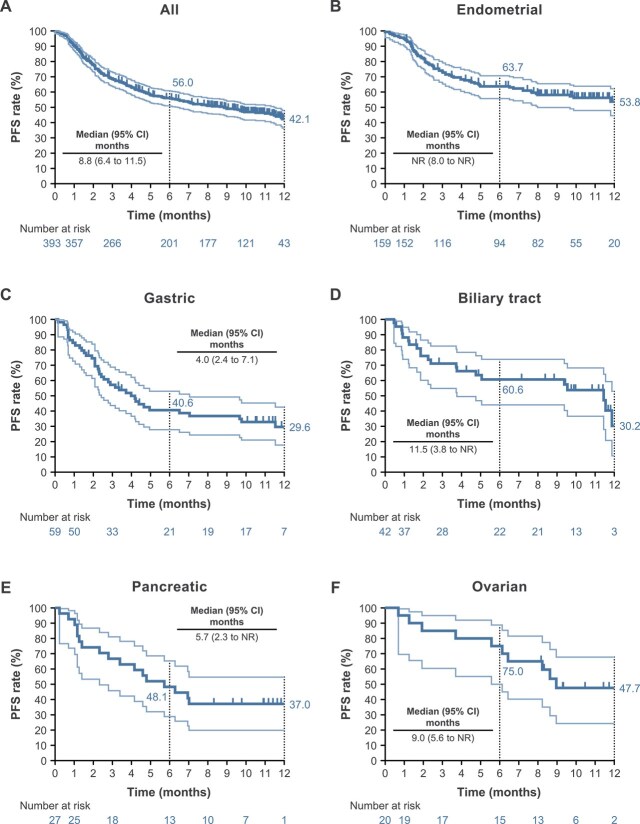
Kaplan–Meier estimates of PFS in all patients (**A**) and those with endometrial (**B**), gastric (**C**), biliary tract (**D**), pancreatic (**E**), and ovarian (**F**) tumors (safety analysis set). Light blue lines indicate 95% CIs and dark blue lines indicate the median. Three patients with no reported clinical responses were excluded from the analysis of PFS because their disease progression during the observation period could not be determined due to their withdrawal from the study. Abbreviations: CI, confidence interval. NR, not reached. PFS, progression-free survival.

**Figure 3:**
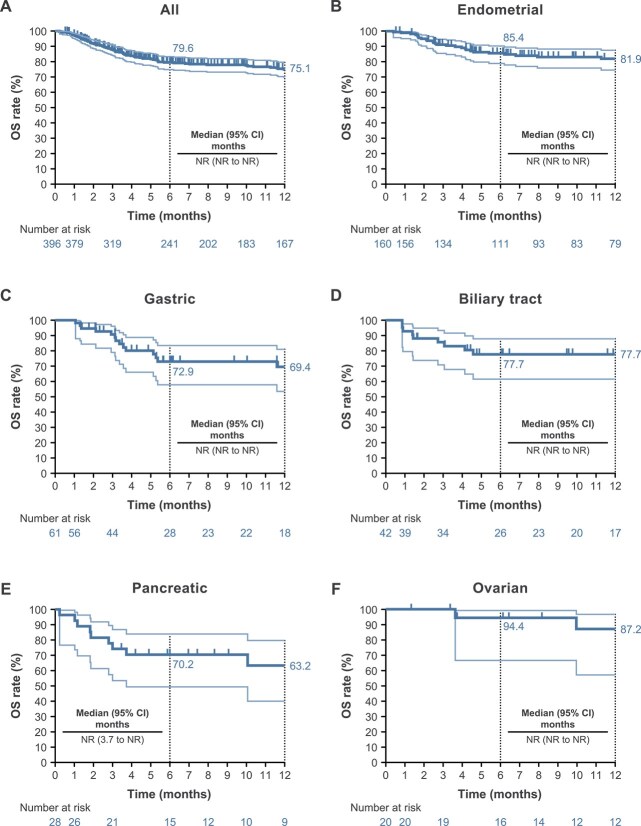
Kaplan–Meier estimates of OS in all patients (**A**) and those with endometrial (**B**), gastric (**C**), biliary tract (**D**), pancreatic (**E**), and ovarian (**F**) tumors (safety analysis set). Light blue lines indicate 95% CIs and dark blue lines indicate the median. Abbreviations: CI, confidence interval. NR, not reached. OS, overall survival.

Three patients with no reported clinical responses were excluded from the analysis of PFS because their disease progression during the observation period could not be determined due to their withdrawal from the study. The Kaplan–Meier curves of PFS and OS in all patients and by tumor type (for tumors that occurred in ≥20 cases) are shown in Figs 2 and 3, respectively. The 6- and 12-month PFS rates were 56.0% and 42.1%, respectively, in all patients, and the respective rates by tumor type are shown in Fig. 2B–F. The median PFS was 8.8 months (95% CI, 6.4–11.5) in all patients and was not reached in those with endometrial cancer. The 6- and 12-month OS rates were 79.6% and 75.1%, respectively, in all patients. The respective rates by tumor type are shown in Fig. 3B–F. The median OS was not reached in all patients and all cancer types.

In patients with endometrial cancer, the 6-month PFS rates were 64.2% in those who received one line of prior therapy and 62.9% in those who received ≥2 lines of prior therapy, and the respective 12-month PFS rates were 55.4% and 51.2%. Additionally, the 6-month OS rates were 88.0% in those who received one line of prior therapy and 84.4% in those who received ≥2 lines of prior therapy, and the respective 12-month OS rates were 82.4% and 81.7%.

In patients with endometrial cancer, 6- and 12-month PFS and OS rates were higher in patients without distant metastasis than in those with distant metastasis (Supplementary Fig. S1), and in patients with a history of radiation therapy vs those without a history of radiation therapy (Supplementary Fig. S2).

#### Safety

Treatment-related AEOSI that occurred in all patients are summarized in Table 3, and details of Grade ≥ 3 AEOSI are summarized in Supplementary Table S7. Among all patients in the safety analysis set (*N* = 396), treatment-related AEOSI of any grade occurred in 128 (32.3%) patients, and those of Grade ≥ 3 occurred in 54 (13.6%) patients. The most frequent AEOSI (any grade) that occurred at a frequency of ≥2% were thyroid dysfunction in 56 patients (14.1%), liver dysfunction/cholangitis sclerosing in 26 (6.6%), colitis/severe diarrhea in 17 (4.3%), interstitial lung disease in 15 (3.8%), and adrenal dysfunction in 11 (2.8%).

**Table 3:** Summary of treatment-related AEOSI in all patients (safety analysis set; *N* = 396).

**Treatment-related AEOSI**	**Any grade**		**Grade ≥3**	
Any AEOSI	128	(32.3)	54	(13.6)
Endocrine disorder	63	(15.9)	9	(2.3)
Pituitary dysfunction	3	(0.8)	0	
Thyroid dysfunction	56	(14.1)	5	(1.3)
Adrenal dysfunction	11	(2.8)	4	(1.0)
Liver dysfunction/cholangitis sclerosing	26	(6.6)	11	(2.8)
Colitis/severe diarrhea	17	(4.3)	11	(2.8)
Interstitial lung disease	15	(3.8)	8^a^	(2.0)
Type 1 diabetes	7	(1.8)	4	(1.0)
Encephalitis/meningitis	5	(1.3)	4	(1.0)
Severe skin reaction^b^	5	(1.3)	3	(0.8)
Infusion reaction	5	(1.3)	3	(0.8)
Renal impairment (e.g. tubulointerstitial nephritis, glomerulonephritis)	4	(1.0)	2	(0.5)
Neuropathy (e.g. Guillain–Barré syndrome)	4	(1.0)	1	(0.3)
Pancreatitis	2	(0.5)	2	(0.5)
Uveitis	1	(0.3)	0	
Myositis/rhabdomyolysis	1	(0.3)	0	
Myasthenia gravis	1	(0.3)	0	
Immune thrombocytopenic purpura	1	(0.3)	0	
Pure red cell aplasia	1	(0.3)	0	

^a^One Grade 5 AE occurred in one patient with esophageal cancer.

^b^For example, toxic epidermal necrolysis, oculomucocutaneous syndrome, erythema multiforme, and pemphigoid.

Data are *n* (%).

Abbreviation: AEOSI, adverse events of special interest.

Of note, one Grade 5 AE occurred in a 63-year-old male patient with esophageal cancer (histological type: moderately differentiated squamous cell carcinoma; stage IV [lung and bone metastases]; complication: chronic kidney disease; ECOG PS of 1 at the start of administration; with history of chest irradiation; smoking status: current smoker). At 22 days after starting treatment, this patient was withdrawn from treatment because of worsening condition (pleural effusion and pleural dissemination). At 28 days after starting treatment, the patient developed interstitial pneumonia (respiratory failure), and died 36 days after starting treatment due to respiratory failure.

**Figure 4:**
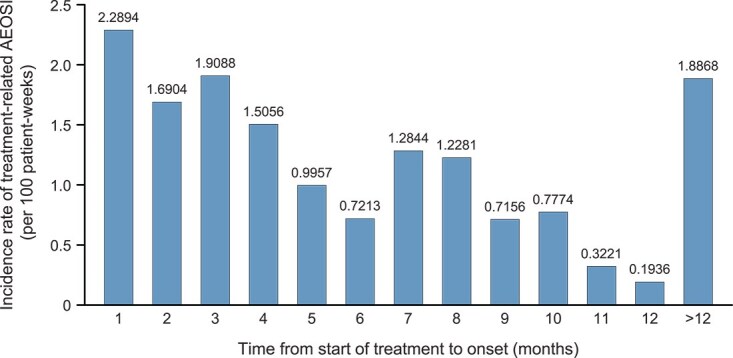
Incidence rate of treatment-related AEOSI according to time from start of treatment to onset (safety analysis set). If more than one AE/reaction occurred or the same event occurred more than once in a single case, these events were compiled into one case. Incidence rate of treatment-related AEOSI = number of cases with events/number of cases in the safety analysis set × 100. AEs that occurred more than 30 days after the final day of administration of pembrolizumab were not included in the compilation. Observed person-days: The administration periods (days) were summed up in the entire analysis set. Incidence (/100 person-weeks): Number of onsets in Week N of administration (observed person-days/7/100). Week 1 of administration was defined as Day 1 to Day 7 of administration and Day ≥358 was handled as Week ≥52 in the compilation. Month unit: One month of administration was defined as Day 1 to Day 30 of administration and Days >360 were handled as >12 months in the compilation. Abbreviation: AEOSI, adverse events of special interest.

**Figure 5:**
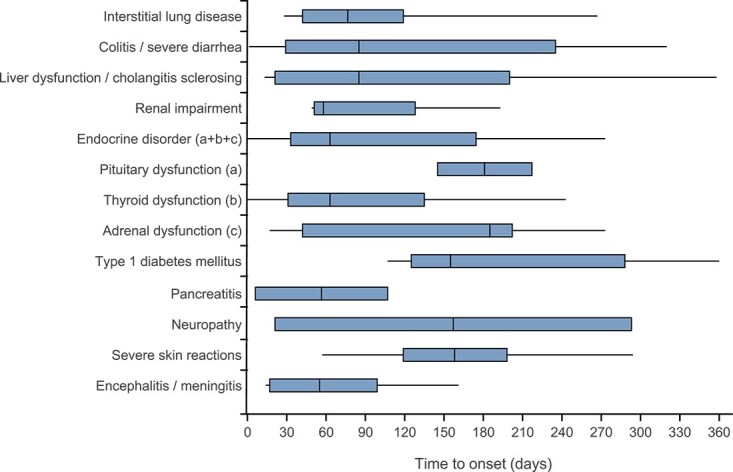
Box and whisker plot of time to onset of treatment-related AEOSI (safety analysis set). Vertical line: median; box: Q1–Q3; whiskers: range (min–max). Uveitis, myositis/rhabdomyolysis, myasthenia gravis, immune thrombocytopenic purpura, and pure red cell aplasia occurred in one patient each. The number of days to the onset of events were as follows: uveitis: 215 days; myositis/rhabdomyolysis: 189 days; myasthenia gravis: 91 days; immune thrombocytopenic purpura: 296 days; and pure red cell aplasia: 115 days. Infusion reaction: Four cases occurred within 1 day, and in one case, the days to the event onset were unknown. Abbreviations: AEOSI, adverse events of special interest. Q, quartile.

After starting treatment, the incidence rates (per 100 patient-weeks) of treatment-related AEOSI showed a decreasing trend from 1 to 12 months (Fig. 4). AEOSI continued to occur >12 months after the start of treatment with pembrolizumab. Figure 5 shows a wide range in times to onset for each treatment-related AEOSI.

The results of AEOSI by tumor type are summarized in Supplementary Table S8. The most frequently reported AEOSI of any grade were thyroid dysfunction (14.4%) and liver dysfunction/cholangitis sclerosing and interstitial lung disease (5.0% each) among patients with endometrial cancer; thyroid dysfunction (11.5%) and liver dysfunction/cholangitis sclerosing (6.6%) among those with gastric cancer; thyroid dysfunction (21.4%), liver dysfunction/cholangitis sclerosing (11.9%), and interstitial lung disease (7.1%) among those with biliary tract cancer; thyroid dysfunction, colitis/severe diarrhea, and type 1 diabetes (7.1% each) among those with pancreatic cancer; and thyroid dysfunction (20.0%), adrenal dysfunction, liver dysfunction/cholangitis sclerosing, and infusion reaction (10.0% each), and pituitary dysfunction and colitis/severe diarrhea (5.0% each) among patients with ovarian cancer.

## Discussion

The present post-marketing surveillance aimed to assess the real-world effectiveness and safety of pembrolizumab monotherapy in Japanese patients with MSI-H solid tumors except for colorectal cancer. Data for approximately 400 cases were collected for this post-marketing surveillance, which represents a notably larger sample size with various tumor types than that of previous studies of pembrolizumab. This study also analyzed data from Japanese patients only, and this is the largest amount of data collected and analyzed to evaluate outcomes of pembrolizumab monotherapy specifically in Japanese patients with MSI-H tumors, as well as in patients with endometrial cancer, compared with KEYNOTE-158, which included only seven Japanese patients with MSI-H tumors.

A total of 403 Japanese patients with MSI-H solid tumors (30 cancer types) were included in this post-marketing surveillance. The most frequently registered cancers were endometrial cancer, gastric cancer, biliary tract cancer, pancreatic cancer, and ovarian cancer. The ORR was 50.3% (CR, 14.1%; PR, 36.2%), the 12-month PFS rate was 42.1%, and the 12-month OS rate was 75.1%. Treatment-related AEOSI of any grade were reported in 128 patients (32.3%) and treatment-related Grade ≥3 AEOSI were reported in 54 patients (13.6%). A Grade 5 AE occurred in one patient with esophageal cancer. No new safety signals were observed.

The overall effectiveness of pembrolizumab in all patients tended to be better in the present study compared with KEYNOTE-158 [10]. The ORR was 50.3% in this study compared with 34.3% in KEYNOTE-158; median PFS was 8.8 months compared with 4.1 months, respectively; and median OS was not reached compared with 23.5 months, respectively. A possible reason for the better OS trend in the present study may be the study design (12-month observation period). Differences in the characteristics of the study populations may have also contributed to the difference in the results between the two studies. For example, the proportion of patients with endometrial cancer was approximately 20% in KEYNOTE-158, compared with approximately 40% in the present study. The reason for the higher enrollment of patients with endometrial cancer may be the high prevalence of MSI-H endometrial cancer in Japan and other countries [5,6,18], as well as the limited treatment options for this cancer type.

The difference in patient background characteristics and proportions of cancer types should be noted when interpreting the overall data. We speculate that one reason the proportions of cancer types in the present study differed from those of KEYNOTE-158 is that MSI-H tests and drug prescriptions were actively performed in patients with cancer types with limited treatment options in Japan (e.g. endometrial cancer and pancreatic cancer).

When focusing on patients with endometrial cancer, the results of the present study were similar to those of KEYNOTE-158 [19]. The slow initial drop in the Kaplan–Meier curve of PFS compared with KEYNOTE-158 may have been because of variations in the timing of efficacy evaluation in clinical practice. As in KEYNOTE-158 and real-world data [20], the efficacy of pembrolizumab alone was confirmed regardless of the number of lines of prior chemotherapy. Although pembrolizumab was used in many patients whose platinum-free interval was less than 6 months, the efficacy of pembrolizumab alone was confirmed regardless of the platinum-free interval.

When focusing on patients with gastric cancer, the results of the present study were similar to those of previous reports with limited numbers of cases, including KEYNOTE-059, KEYNOTE-061, and KEYNOTE-062. The ORR was 40.7% in this study (*n* = 61), which is consistent with the ORR of 45.8% in KEYNOTE-158 (*n* = 24) [10], 57.1% in KEYNOTE-059 (*n* = 7 including one Japanese patient) [21], 46.7% in KEYNOTE-061 (*n* = 15 including two Japanese patients) [21], and 57.1% in KEYNOTE-062 (*n* = 14, combined positive score ≥ 1, including three Japanese patients) [21,22].

Regarding ovarian cancer, although the effectiveness results appeared to be better in the present study vs KEYNOTE-158 [12], this may have been because of the smaller sample size in the present study vs KEYNOTE-158 (20 vs 24, respectively). Another reason may be related to differences in histological type in Japanese and non-Japanese patients. Among Japanese patients with ovarian cancer, the prevalence of clear cell carcinoma is higher compared with that among Western patients [23,24]. Although complete histologic data were not obtained for this post-marketing surveillance, there were six patients with ovarian clear cell carcinoma in this study (the ORR was 100% among the six patients with ovarian clear cell carcinoma), which is more responsive to immune checkpoint inhibitors than other histologic types [25–29]. Although the prevalence of clear cell carcinoma is higher in Japanese vs Western patients [23,24], its sensitivity to immune checkpoint inhibitors is not Japanese-specific.

No new safety concerns were raised in this post-marketing surveillance. Although some severe treatment-related AEOSI were observed, the frequency was low.

The present study has some limitations, including those inherent to the observational study design and population bias caused by tumor type and treatment history. The duration of treatment and follow-up (1 year) was too short to be able to draw meaningful comparisons with the updated analysis of KEYNOTE-158, which had a median time from the first dose to database cutoff of 37.5 months [12]. Safety and effectiveness were assessed by each investigator (e.g. tumor response evaluation was not centralized), and effectiveness assessment time points were not specified and varied between physicians (evaluation occurred during daily medical practice). Some events (death and PD) after discontinuation of pembrolizumab may not have been reported. Finally, AEs were collected within only 30 days of the final dose, which may have led to an underestimation of late-onset toxicities, including immune-related AEs. However, as this was a post-marketing surveillance study conducted within the framework of routine clinical practice rather than a controlled clinical trial, data collection was based on real-world monitoring rather than predefined protocols.

In conclusion, this post-marketing surveillance confirmed the real-world effectiveness and safety of pembrolizumab monotherapy in patients with MSI-H solid tumors except colorectal cancer in Japan. While some regimens may be used without biomarker testing, particularly in gynecologic and gastrointestinal malignancies such as endometrial and gastric cancers, MSI/MMR testing remains essential to guide treatment decisions. Given the limited data on non-colorectal MSI-H tumors, our study provides valuable clinical insights. Further research is needed to optimize treatment strategies in this patient population.

## Supplementary Material

Supplementary_Material_hyaf064

## Data Availability

The data sets analyzed during this post-marketing surveillance are not available because data sharing with third parties is not permitted per the contract with all study sites or the patients. Please contact MSD K.K., Tokyo, Japan (https://www.msd.co.jp) for inquiries about access to the data set used in this post-marketing surveillance.
