# Supplementary material for: Effectiveness and safety of pembrolizumab for the treatment of Japanese patients with microsatellite instability-high tumors excluding colorectal cancer: a post-marketing surveillance
Source: Jpn J Clin Oncol. 2025 May 18;55(8):901–12. doi: 10.1093/jjco/hyaf064 (PMC12319222; doi:10.1093/jjco/hyaf064)
Supplement: Supplementary_Material_hyaf064 [file supplementary_material_hyaf064.docx]

# Supplementary Material

**Supplementary Table S1.** Tumor types (primary diagnosis) (Entire enrolled patient population; *N* = 403)

| **Tumor type** | ***n* (%)** | **Tumor type** | ***n* (%)** |
| --- | --- | --- | --- |
| Endometrial cancer | 162 (40.2) | Glioblastoma | 3 (0.7) |
| Gastric cancer | 61 (15.1) | Small cell lung cancer | 2 (0.5) |
| Biliary tract cancer | 42 (10.4) | Neuroendocrine carcinoma | 2 (0.5) |
| Pancreatic cancer | 29 (7.2) | Paget’s disease | 1 (0.2) |
| Ovarian cancer^a^ | 20 (5.0) | Ewing sarcoma | 1 (0.2) |
| Small intestinal cancer^b^ | 16 (4.0) | Pheochromocytoma | 1 (0.2) |
| Uterine carcinosarcoma | 11 (2.7) | Teratoma | 1 (0.2) |
| Cervical cancer | 10 (2.5) | Thymic carcinoma | 1 (0.2) |
| Prostate cancer | 9 (2.2) | Angiosarcoma | 1 (0.2) |
| Breast cancer | 7 (1.7) | Primary extragonadal germ cell tumor | 1 (0.2) |
| Unknown primary | 7 (1.7) | Appendix cancer | 1 (0.2) |
| Esophageal cancer | 6 (1.5) | Sarcoma | 1 (0.2) |
| Hepatocellular cancer | 4 (1.0) | Nasal cavity cancer | 1 (0.2) |
| Uterine sarcoma | 3 (0.7) | Leiomyosarcoma | 1 (0.2) |
| Neuroendocrine tumor | 3 (0.7) | Choriocarcinoma | 1 (0.2) |

^a^Including two patients with peritoneal cancer.

^b^One case of small intestinal cancer was duodenal cancer.

Some patients were counted in multiple categories.

**Supplementary Table S2.** Baseline demographic and clinical characteristics of all patients and those with endometrial, gastric, biliary tract, pancreatic, and ovarian tumors (safety analysis set)

|  | **All**  ***N* = 396** | **Endometrial**  ***N* = 160** | **Gastric**  ***N* = 61** | **Biliary tract**  ***N* = 42** | **Pancreatic**  ***N* = 28** | **Ovarian**  ***N* = 20^a^** |
| --- | --- | --- | --- | --- | --- | --- |
| Sex |  |  |  |  |  |  |
| Male | 112 (28.3) | 0 | 35 (57.4) | 23 (54.8) | 16 (57.1) | 0 |
| Female | 284 (71.7) | 160 (100) | 26 (42.6) | 19 (45.2) | 12 (42.9) | 20 (100.0) |
| Age, median (range), years | 63 (34–96) | 58 (38–81) | 72 (36–96) | 69 (42–81) | 69 (39–84) | 49 (34–74) |
| <40 years | 12 (3.0) | 1 (0.6) | 2 (3.3) | 0 | 1 (3.6) | 5 (25.0) |
| 40–49 years | 50 (12.6) | 20 (12.5) | 3 (4.9) | 1 (2.4) | 3 (10.7) | 5 (25.0) |
| 50–59 years | 101 (25.5) | 65 (40.6) | 3 (4.9) | 7 (16.7) | 0 | 5 (25.0) |
| 60–69 years | 120 (30.3) | 50 (31.3) | 16 (26.2) | 14 (33.3) | 11 (39.3) | 3 (15.0) |
| 70–79 years | 88 (22.2) | 20 (12.5) | 24 (39.3) | 17 (40.5) | 11 (39.3) | 2 (10.0) |
| ≥80 years | 23 (5.8) | 2 (1.3) | 13 (21.3) | 3 (7.1) | 2 (7.1) | 0 |
| Unknown | 2 (0.5) | 2 (1.3) | 0 | 0 | 0 | 0 |
| ECOG PS |  |  |  |  |  |  |
| 0 | 206 (52.0) | 95 (59.4) | 30 (49.2) | 23 (54.8) | 16 (57.1) | 11 (55.0) |
| 1 | 133 (33.6) | 40 (25.0) | 25 (41.0) | 15 (35.7) | 7 (25.0) | 7 (35.0) |
| 2 | 33 (8.3) | 12 (7.5) | 6 (9.8) | 2 (4.8) | 3 (10.7) | 1 (5.0) |
| 3 | 22 (5.6) | 13 (8.1) | 0 | 2 (4.8) | 2 (7.1) | 1 (5.0) |
| 4 | 2 (0.5) | 0 | 0 | 0 | 0 | 0 |
| Distant metastasis |  |  |  |  |  |  |
| No | 75 (18.9) | 36 (22.5) | 5 (8.2) | 8 (19.0) | 6 (21.4) | 4 (20.0) |
| Yes | 321 (81.1) | 124 (77.5) | 56 (91.8) | 34 (81.0) | 22 (78.6) | 16 (80.0) |
| Distant lymph nodes | 135 (34.1) | 44 (27.5) | 32 (52.5) | 13 (31.0) | 12 (42.9) | 7 (35.0) |
| Lung | 87 (22.0) | 42 (26.3) | 4 (6.6) | 6 (14.3) | 6 (21.4) | 6 (30.0) |
| Liver | 81 (20.5) | 18 (11.3) | 17 (27.9) | 16 (38.1) | 11 (39.3) | 4 (20.0) |
| Prior pre-operative/post-operative chemotherapy | 176 (44.4) | 103 (64.4) | 17 (27.9) | 9 (21.4) | 8 (28.6) | 16 (80.0) |
| Prior lines of chemotherapy |  |  |  |  |  |  |
| 0 | 5 (1.3) | 2 (1.3)^b^ | 0 | 0 | 1 (3.6) | 0 |
| 1 | 154 (38.9) | 54 (33.8)^b^ | 23 (37.7) | 15 (35.7) | 10 (35.7) | 5 (25.0) |
| 2 | 116 (29.3) | 66 (41.3)^b^ | 19 (31.1) | 20 (47.6) | 11 (39.3) | 7 (35.0) |
| 3 | 38 (9.6) | 22 (13.8)^b^ | 6 (9.8) | 3 (7.1) | 3 (10.7) | 2 (10.0) |
| ≥4 | 40 (10.1) | 14 (8.8)^b^ | 8 (13.1) | 2 (4.8) | 3 (10.7) | 3 (15.0) |
| Unknown | 5 (1.3) | 2 (1.3)^b^ | 1 (1.6) | 1 (2.4) | 0 | 0 |

Data are *n* (%) unless otherwise stated.

^a^Including two patients with peritoneal cancer.

^b^For patients with endometrial cancer, pre-operative/post-operative chemotherapy was included as prior lines of therapy.

Abbreviation: ECOG PS, Eastern Cooperative Oncology Group performance status.

**Supplementary Table S3.** Baseline demographic and clinical characteristics of all patients and those with endometrial, gastric, biliary tract, pancreatic, and ovarian tumors (effectiveness analysis set)

|  | **All**  ***N* = 376** | **Endometrial**  ***N* = 155** | **Gastric**  ***N* = 54** | **Biliary tract**  ***N* = 40** | **Pancreatic**  ***N* = 25** | **Ovarian**  ***N* = 20^a^** |
| --- | --- | --- | --- | --- | --- | --- |
| Sex |  |  |  |  |  |  |
| Male | 103 (27.4) | 0 | 31 (57.4) | 21 (52.5) | 15 (60.0) | 0 |
| Female | 273 (72.6) | 155 (100.0) | 23 (42.6) | 19 (47.5) | 10 (40.0) | 20 (100.0) |
| Age, median (range), years | 62 (34–96) | 58 (38–81) | 72 (36–96) | 68 (42–81) | 69 (39–81) | 49 (34–74) |
| <40 years | 12 (3.2) | 1 (0.6) | 2 (3.7) | 0 | 1 (4.0) | 5 (25.0) |
| 40–49 years | 47 (12.5) | 20 (12.9) | 2 (3.7) | 1 (2.5) | 3 (12.0) | 5 (25.0) |
| 50–59 years | 98 (26.1) | 62 (40.0) | 3 (5.6) | 7 (17.5) | 0 | 5 (25.0) |
| 60–69 years | 116 (30.9) | 49 (31.6) | 16 (29.6) | 14 (35.0) | 10 (40.0) | 3 (15.0) |
| 70–79 years | 82 (21.8) | 19 (12.3) | 20 (37.0) | 16 (40.0) | 10 (40.0) | 2 (10.0) |
| ≥80 years | 19 (5.1) | 2 (1.3) | 11 (20.4) | 2 (5.0) | 1 (4.0) | 0 |
| Unknown | 2 (0.5) | 2 (1.3) | 0 | 0 | 0 | 0 |
| ECOG PS |  |  |  |  |  |  |
| 0 | 202 (53.7) | 94 (60.6) | 28 (51.9) | 22 (55.0) | 15 (60.0) | 11 (55.0) |
| 1 | 123 (32.7) | 39 (25.2) | 20 (37.0) | 14 (35.0) | 6 (24.0) | 7 (35.0) |
| 2 | 30 (8.0) | 11 (7.1) | 6 (11.1) | 2 (5.0) | 2 (8.0) | 1 (5.0) |
| 3 | 19 (5.1) | 11 (7.1) | 0 | 2 (5.0) | 2 (8.0) | 1 (5.0) |
| 4 | 2 (0.5) | 0 | 0 | 0 | 0 | 0 |
| Distant metastasis |  |  |  |  |  |  |
| No | 74 (19.7) | 36 (23.2) | 5 (9.3) | 8 (20.0) | 6 (24.0) | 4 (20.0) |
| Yes | 302 (80.3) | 119 (76.8) | 49 (90.7) | 32 (80.0) | 19 (76.0) | 16 (80.0) |
| Distant lymph nodes | 129 (34.3) | 42 (27.1) | 29 (53.7) | 12 (30.0) | 11 (44.0) | 7 (35.0) |
| Lung | 80 (21.3) | 40 (25.8) | 4 (7.4) | 5 (12.5) | 4 (16.0) | 6 (30.0) |
| Liver | 75 (19.9) | 16 (10.3) | 16 (29.6) | 15 (37.5) | 11 (44.0) | 4 (20.0) |
| Prior pre-operative/post-operative chemotherapy | 169 (44.9) | 100 (64.5) | 16 (29.6) | 9 (22.5) | 6 (24.0) | 16 (80.0) |
| Prior lines of chemotherapy |  |  |  |  |  |  |
| 0 | 5 (1.3) | 2 (1.3)^b^ | 0 | 0 | 1 (4.0) | 0 |
| 1 | 148 (39.4) | 53 (34.2)^b^ | 21 (38.9) | 14 (35.0) | 8 (32.0) | 5 (25.0) |
| 2 | 109 (29.0) | 63 (40.6)^b^ | 17 (31.5) | 19 (47.5) | 11 (44.0) | 7 (35.0) |
| 3 | 36 (9.6) | 22 (14.2)^b^ | 5 (9.3) | 3 (7.5) | 3 (12.0) | 2 (10.0) |
| ≥4 | 36 (9.6) | 13 (8.4)^b^ | 6 (11.1) | 2 (5.0) | 2 (8.0) | 3 (15.0) |
| Unknown | 5 (1.3) | 2 (1.3)^b^ | 1 (1.9) | 1 (2.5) | 0 | 0 |

Data are *n* (%) unless otherwise stated.

^a^Including two patients with peritoneal cancer.

^b^For patients with endometrial cancer, pre-operative/post-operative chemotherapy was included as prior lines of therapy.

Abbreviation: ECOG PS, Eastern Cooperative Oncology Group performance status.

**Supplementary Table S4.** MSI testing (safety analysis set)

|  | **All**  ***N* = 396** | **Endometrial**  ***N* = 160** | **Gastric**  ***N* = 61** | **Biliary tract**  ***N* = 42** | **Pancreatic**  ***N* = 28** | **Ovarian**  ***N* = 20^a^** |
| --- | --- | --- | --- | --- | --- | --- |
| MSI testing method |  |  |  |  |  |  |
| PCR | 358 (90.4) | 143 (89.4) | 56 (91.8) | 37 (88.1) | 26 (92.9) | 17 (85.0) |
| IHC | 1 (0.3) | 1 (0.6) | 0 | 0 | 0 | 0 |
| Unknown | 37 (9.3) | 16 (10.0) | 5 (8.2) | 5 (11.9) | 2 (7.1) | 3 (15.0) |
| Sample type |  |  |  |  |  |  |
| Biopsy | 140 (35.4) | 28 (17.5) | 36 (59.0) | 24 (57.1) | 16 (57.1) | 4 (20.0) |
| Surgical resection sample | 251 (63.4) | 130 (81.3) | 25 (41.0) | 18 (42.9) | 12 (42.9) | 16 (80.0) |
| Unknown | 5 (1.3) | 2 (1.3) | 0 | 0 | 0 | 0 |
| Sample location |  |  |  |  |  |  |
| Primary site | 331 (83.6) | 131 (81.9) | 58 (95.1) | 35 (83.3) | 23 (82.1) | 16 (80.0) |
| Metastatic site | 57 (14.4) | 26 (16.3) | 2 (3.3) | 7 (16.7) | 5 (17.9) | 4 (20.0) |
| Both primary and metastatic | 1 (0.3) | 1 (0.6) | 0 | 0 | 0 | 0 |
| Unknown | 7 (1.8) | 2 (1.3) | 1 (1.6) | 0 | 0 | 0 |
| Time from MSI-H diagnosis to start of treatment^b^ | *n* = 349 | *n* = 141 | *n* = 53 | *n* = 37 | *n* = 23 | *n* = 17 |
| Median (range), weeks | 3.9 (0.1–40.9) | 4 (0.3–40.9) | 4.4 (1.0–25.1) | 3.9 (0.1–24.0) | 2.6 (0.3–11.9) | 4.1 (0.6–21.1) |

Data are *n* (%) unless otherwise stated.

^a^Including two patients with peritoneal cancer.

^b^Data collected from December 2018 after approval of companion diagnostic.

Abbreviations: IHC, immunohistochemistry. MSI, microsatellite instability. MSI-H, high-frequency MSI. PCR, polymerase chain reaction.

**Supplementary Table S5.** Duration from prior chemotherapy to start of treatment in patients with endometrial cancer

|  | **Effectiveness analysis set^a^** |
| --- | --- |
|  | ***N* = 152** |
| Duration, median (range), months | 3 (0–50) |
| <6 months | 100 (65.8) |
| 6 to <12 months | 30 (19.7) |
| ≥12 months | 21 (13.8) |
| Unknown | 1 (0.7) |

Data are *n* (%) unless otherwise stated.

^a^Excluding two patients with no prior platinum chemotherapy and one patient with unknown prior chemotherapy, including pre-operative/post-operative chemotherapy.

**Supplementary Table S6.** Response outcome of endometrial cancer by platinum-free interval (effectiveness analysis set)

| **Total** | *N* = 152^a^ |
| --- | --- |
| Objective response | 86 (56.6) |
| Disease control | 112 (73.7) |
| Complete response | 33 (21.7) |
| Partial response | 53 (34.9) |
| **PFI <6 months** | *n* = 100 |
| Objective response | 57 (57.0) |
| Disease control | 70 (70.0) |
| Complete response | 22 (22.0) |
| Partial response | 35 (35.0) |
| **PFI 6 to <12 months** | *n* = 29 |
| Objective response | 17 (58.6) |
| Disease control | 23 (79.3) |
| Complete response | 6 (20.7) |
| Partial response | 11 (37.9) |
| **PFI ≥12 months** | *n* = 21 |
| Objective response | 10 (47.6) |
| Disease control | 17 (81.0) |
| Complete response | 3 (14.3) |
| Partial response | 7 (33.3) |

Data are *n* (%).

^a^Including two patients who did not receive prior platinum therapy.

Abbreviation: PFI, platinum-free interval.

**Supplementary Table S7.** Details of Grade ≥3 adverse events of special interest (safety analysis set)

|  | **Cancer type** | **Sex** | **Age** | **Event** | **Time to onset, days** | **Grade** | **Outcome** | **Time to recovery, days** | **Treatment** |
| --- | --- | --- | --- | --- | --- | --- | --- | --- | --- |
| 1 | Esophageal | M | 63 | Pleural effusion and pleural dissemination  Respiratory failure | 22  28 | 5 | Death |  | Drug withdrawn |
| 2 | Endometrial | F | 38 | Autoimmune pancreatitis | 6 | 4 | Recovering |  | Drug withdrawn |
| 3 | Endometrial | F | 44 | Interstitial lung disease | 55 | 4 | Recovering |  | Not applicable^a^ |
| 4 | Endometrial | F | 46 | Diabetic ketoacidosis | 107 | 4 | Recovered | 34 | Drug interrupted |
| 5 | Endometrial | F | 56 | Meningitis aseptic | 14 | 4 | Recovered | 77 | Drug withdrawn |
| 6 | Endometrial | F | 58 | Fulminant type 1 diabetes mellitus | 240 | 4 | Recovering |  | Drug interrupted |
| 7 | Endometrial | F | 62 | Thyroiditis | 63 | 4 | Recovering |  | Drug interrupted |
| 8 | Endometrial | F | 62 | Fulminant type 1 diabetes mellitus | 148 | 4 | Recovered | 26 | Drug withdrawn |
| 9 | Endometrial | F | 70 | Interstitial lung disease | 213 | 4 | Recovering |  | Drug withdrawn |
| 10 | Endometrial | F | 53 | Autoimmune thyroiditis | 76 | 3 | Unknown |  | Drug withdrawn |
| 11 | Endometrial | F | 55 | Immune-mediated hepatic disorder | 217 | 3 | Recovered | 1 | Drug withdrawn |
| 12 | Endometrial | F | 55 | Diarrhea | 39 | 3 | Recovering |  | Drug interrupted |
| 13 | Endometrial | F | 56 | Immune-mediated encephalitis | 99 | 3 | Recovering |  | Drug withdrawn |
| 14 | Endometrial | F | 58 | Interstitial lung disease | 31 | 3 | Recovering |  | Not applicable^a^ |
| 15 | Endometrial | F | 61 | Hypoalbuminemia | 301 | 3 | Recovered |  | Not applicable^a^ |
| 16 | Endometrial | F | 64 | Immune-mediated pancreatitis | 107 | 3 | Recovering |  | Drug interrupted |
| 17 | Endometrial | F | 64 | Stevens-Johnson syndrome | 198 | 3 | Recovering |  | Not applicable^a^ |
|  |  |  |  | Erythema multiforme | 198 | 3 | Recovering |  | Not applicable^a^ |
| 18 | Endometrial | F | 66 | Diarrhea | - | 3 | Recovering |  | Dose not changed |
|  |  |  |  | Diarrhea | 6 | 3 | Recovered | 11 | Dose not changed |
|  |  |  |  | Diarrhea | 50 | 3 | Recovered | 5 | Dose not changed |
|  |  |  |  | Diarrhea | 133 | 3 | Recovered | 2 | Dose not changed |
|  |  |  |  | Diarrhea | 140 | 3 | Recovered | 3 | Dose not changed |
| 19 | Endometrial | F | 67 | Hepatic function abnormal | 158 | 3 | Recovered | 32 | Drug withdrawn |
|  |  |  |  | Erythema multiforme | 158 | 3 | Recovered | 16 | Drug withdrawn |
| 20 | Endometrial | F | 67 | Adrenal insufficiency | 202 | 3 | Recovering |  | Drug withdrawn |
| 21 | Endometrial | F | 67 | Thyroiditis | 211 | 3 | Recovering |  | Dose not changed |
| 22 | Endometrial | F | 69 | Diarrhea | 192 | 3 | Recovered | 433 | Drug withdrawn |
| 23 | Endometrial | F | 69 | Infusion-related reaction | 1 | 3 | Recovered | 5 | Dose not changed |
| 24 | Biliary tract | M | 71 | Interstitial lung disease | 102 | 4 | Recovering |  | Drug withdrawn |
| 25 | Biliary tract | F | 51 | Immune-mediated hepatitis | 21 | 3 | Recovered | 50 | Drug withdrawn |
| 26 | Biliary tract | M | 61 | Tubulointerstitial nephritis | 49 | 3 | Recovering |  | Unknown |
| 27 | Biliary tract | M | 63 | Interstitial lung disease | 267 | 3 | Recovered | 27 | Drug interrupted |
| 28 | Biliary tract | M | 68 | Adrenal insufficiency | 195 | 3 | Recovering |  | Drug withdrawn |
| 29 | Biliary tract | F | 73 | Thyroid disorder | 170 | 3 | Recovering |  | Drug withdrawn |
| 30 | Biliary tract | M | 75 | Hypothyroidism | 133 | 3 | Recovered | 34 | Not applicable^a^ |
| 31 | Biliary tract | M | 78 | Pemphigoid | 294 | 3 | Recovering |  | Drug interrupted |
| 32 | Biliary tract | M | 79 | Interstitial lung disease | 77 | 3 | Recovered | 18 | Drug withdrawn |
| 33 | Gastric | F | 44 | Hepatic function abnormal | 21 | 3 | Recovering |  | Drug interrupted |
| 34 | Gastric | F | 69 | Hepatic function abnormal | 13 | 3 | Recovered | 43 | Drug withdrawn |
| 35 | Gastric | M | 70 | Diarrhea | 15 | 3 | Recovering |  | Drug interrupted |
|  |  |  |  | Hepatic function abnormal | 78 | 3 | Recovering |  | Drug interrupted |
| 36 | Gastric | F | 74 | Hepatic function abnormal | 58 | 3 | Recovering |  | Drug withdrawn |
| 37 | Gastric | M | 84 | Neuropathy peripheral | 21 | 3 | Not recovered |  | Drug withdrawn |
| 38 | Pancreatic | M | 64 | Type 1 diabetes mellitus | 360 | 3 | Recovered | 56 | Drug withdrawn |
| 39 | Ovarian | F | 50 | Infusion-related reaction | 0 | 3 | Recovered | 4 | Drug interrupted |
| 40 | Ovarian | F | 50 | Infusion-related reaction | 1 | 3 | Recovered | 6 | Drug withdrawn |

^a^Adverse event developed after completion of pembrolizumab administration.

Abbreviations: F, female. M, male.

**Supplementary Table S8.** Summary of treatment-related AEOSI in patients with endometrial (**A**), gastric (**B**), biliary tract (**C**), pancreatic (**D**), and ovarian (**E**) tumors (safety analysis set)

**(A) Endometrial (*N* = 160)**

| **Treatment-related AEOSI** | **Any grade** | | **Grade ≥3** | |
| --- | --- | --- | --- | --- |
| Any AEOSI | 55 | (34.4) | 22 | (13.8) |
| Endocrine disorder | 25 | (15.6) | 4 | (2.5) |
| Pituitary dysfunction | 2 | (1.3) | 0 |  |
| Thyroid dysfunction | 23 | (14.4) | 3 | (1.9) |
| Adrenal dysfunction | 4 | (2.5) | 1 | (0.6) |
| Liver dysfunction/cholangitis sclerosing | 8 | (5.0) | 3 | (1.9) |
| Colitis/severe diarrhea | 5 | (3.1) | 3 | (1.9) |
| Interstitial lung disease | 8 | (5.0) | 3 | (1.9) |
| Type 1 diabetes | 4 | (2.5) | 3 | (1.9) |
| Encephalitis/meningitis | 3 | (1.9) | 2 | (1.3) |
| Severe skin reactions^a^ | 2 | (1.3) | 2 | (1.3) |
| Infusion reaction | 2 | (1.3) | 1 | (0.6) |
| Renal impairment (e.g., tubulointerstitial nephritis, glomerulonephritis) | 0 |  | 0 |  |
| Neuropathy (e.g., Guillain–Barré syndrome) | 2 | (1.3) | 0 |  |
| Pancreatitis | 2 | (1.3) | 2 | (1.3) |
| Uveitis | 0 |  | 0 |  |
| Myositis/rhabdomyolysis | 0 |  | 0 |  |
| Myasthenia gravis | 0 |  | 0 |  |
| Immune thrombocytopenic purpura | 0 |  | 0 |  |
| Pure red cell aplasia | 1 | (0.6) | 0 |  |

Data are *n* (%).

^a^E.g., toxic epidermal necrolysis, oculomucocutaneous syndrome, erythema multiforme, pemphigoid.

Abbreviation: AEOSI, adverse events of special interest.

**(B) Gastric (*N* = 61)**

| **Treatment-related AEOSI** | **Any grade** | | **Grade ≥3** | |
| --- | --- | --- | --- | --- |
| Any AEOSI | 17 | (27.9) | 5 | (8.2) |
| Endocrine disorder | 8 | (13.1) | 0 |  |
| Pituitary dysfunction | 0 |  | 0 |  |
| Thyroid dysfunction | 7 | (11.5) | 0 |  |
| Adrenal dysfunction | 1 | (1.6) | 0 |  |
| Liver dysfunction/cholangitis sclerosing | 4 | (6.6) | 4 | (6.6) |
| Colitis/severe diarrhea | 1 | (1.6) | 1 | (1.6) |
| Interstitial lung disease | 0 |  | 0 |  |
| Type 1 diabetes | 0 |  | 0 |  |
| Encephalitis/meningitis | 0 |  | 0 |  |
| Severe skin reactions^a^ | 2 | (3.3) | 0 |  |
| Infusion reaction | 1 | (1.6) | 0 |  |
| Renal impairment (e.g., tubulointerstitial nephritis, glomerulonephritis) | 2 | (3.3) | 0 |  |
| Neuropathy (e.g., Guillain–Barré syndrome) | 1 | (1.6) | 1 | (1.6) |
| Pancreatitis | 0 |  | 0 |  |
| Uveitis | 0 |  | 0 |  |
| Myositis/rhabdomyolysis | 0 |  | 0 |  |
| Myasthenia gravis | 0 |  | 0 |  |
| Immune thrombocytopenic purpura | 1 | (1.6) | 0 |  |
| Pure red cell aplasia | 0 |  | 0 |  |

Data are *n* (%).

^a^E.g., toxic epidermal necrolysis, oculomucocutaneous syndrome, erythema multiforme, pemphigoid.

Abbreviation: AEOSI, adverse events of special interest.

**(C) Biliary tract (*N* = 42)**

| **Treatment-related AEOSI** | **Any grade** | | **Grade ≥3** | |
| --- | --- | --- | --- | --- |
| Any AEOSI | 17 | (40.5) | 9 | (21.4) |
| Endocrine disorder | 10 | (23.8) | 3 | (7.1) |
| Pituitary dysfunction | 0 |  | 0 |  |
| Thyroid dysfunction | 9 | (21.4) | 2 | (4.8) |
| Adrenal dysfunction | 1 | (2.4) | 1 | (2.4) |
| Liver dysfunction/cholangitis sclerosing | 5 | (11.9) | 1 | (2.4) |
| Colitis/severe diarrhea | 0 |  | 0 |  |
| Interstitial lung disease | 3 | (7.1) | 3 | (7.1) |
| Type 1 diabetes | 1 | (2.4) | 0 |  |
| Encephalitis/meningitis | 0 |  | 0 |  |
| Severe skin reactions^a^ | 1 | (2.4) | 1 | (2.4) |
| Infusion reaction | 0 |  | 0 |  |
| Renal impairment (e.g., tubulointerstitial nephritis, glomerulonephritis) | 2 | (4.8) | 1 | (2.4) |
| Neuropathy (e.g., Guillain–Barré syndrome) | 0 |  | 0 |  |
| Pancreatitis | 0 |  | 0 |  |
| Uveitis | 0 |  | 0 |  |
| Myositis/rhabdomyolysis | 0 |  | 0 |  |
| Myasthenia gravis | 1 | (2.4) | 0 |  |
| Immune thrombocytopenic purpura | 0 |  | 0 |  |
| Pure red cell aplasia | 0 |  | 0 |  |

Data are *n* (%).

^a^E.g., toxic epidermal necrolysis, oculomucocutaneous syndrome, erythema multiforme, pemphigoid.

Abbreviation: AEOSI, adverse events of special interest.

**(D) Pancreatic (*N* = 28)**

| **Treatment-related AEOSI** | **Any grade** | | **Grade ≥3** | |
| --- | --- | --- | --- | --- |
| Any AEOSI | 6 | (21.4) | 1 | (3.6) |
| Endocrine disorder | 2 | (7.1) | 0 |  |
| Pituitary dysfunction | 0 |  | 0 |  |
| Thyroid dysfunction | 2 | (7.1) | 0 |  |
| Adrenal dysfunction | 0 |  | 0 |  |
| Liver dysfunction/cholangitis sclerosing | 1 | (3.6) | 0 |  |
| Colitis/severe diarrhea | 2 | (7.1) | 0 |  |
| Interstitial lung disease | 0 |  | 0 |  |
| Type 1 diabetes | 2 | (7.1) | 1 | (3.6) |
| Encephalitis/meningitis | 0 |  | 0 |  |
| Severe skin reactions^a^ | 0 |  | 0 |  |
| Infusion reaction | 0 |  | 0 |  |
| Renal impairment (e.g., tubulointerstitial nephritis, glomerulonephritis) | 0 |  | 0 |  |
| Neuropathy (e.g., Guillain–Barré syndrome) | 0 |  | 0 |  |
| Pancreatitis | 0 |  | 0 |  |
| Uveitis | 0 |  | 0 |  |
| Myositis/rhabdomyolysis | 1 | (3.6) | 0 |  |
| Myasthenia gravis | 0 |  | 0 |  |
| Immune thrombocytopenic purpura | 0 |  | 0 |  |
| Pure red cell aplasia | 0 |  | 0 |  |

Data are *n* (%).

^a^E.g., toxic epidermal necrolysis, oculomucocutaneous syndrome, erythema multiforme, pemphigoid.

Abbreviation: AEOSI, adverse events of special interest.

**(E) Ovarian (*N* = 20)**

| **Treatment-related AEOSI** | **Any grade** | | **Grade ≥3** | |
| --- | --- | --- | --- | --- |
| Any AEOSI | 7 | (35.0) | 2 | (10.0) |
| Endocrine disorder | 4 | (20.0) | 0 |  |
| Pituitary dysfunction | 1 | (5.0) | 0 |  |
| Thyroid dysfunction | 4 | (20.0) | 0 |  |
| Adrenal dysfunction | 2 | (10.0) | 0 |  |
| Liver dysfunction/cholangitis sclerosing | 2 | (10.0) | 0 |  |
| Colitis/severe diarrhea | 1 | (5.0) | 0 |  |
| Interstitial lung disease | 0 |  | 0 |  |
| Type 1 diabetes | 0 |  | 0 |  |
| Encephalitis/meningitis | 0 |  | 0 |  |
| Severe skin reactions^a^ | 0 |  | 0 |  |
| Infusion reaction | 2 | (10.0) | 2 | (10.0) |
| Renal impairment (e.g., tubulointerstitial nephritis, glomerulonephritis) | 0 |  | 0 |  |
| Neuropathy (e.g., Guillain–Barré syndrome) | 0 |  | 0 |  |
| Pancreatitis | 0 |  | 0 |  |
| Uveitis | 0 |  | 0 |  |
| Myositis/rhabdomyolysis | 0 |  | 0 |  |
| Myasthenia gravis | 0 |  | 0 |  |
| Immune thrombocytopenic purpura | 0 |  | 0 |  |
| Pure red cell aplasia | 0 |  | 0 |  |

Data are *n* (%).

^a^E.g., toxic epidermal necrolysis, oculomucocutaneous syndrome, erythema multiforme, pemphigoid.

Abbreviation: AEOSI, adverse events of special interest.

**Supplementary Figure 1.** Kaplan–Meier curves of (**A**) PFS and (**B**) OS in patients with endometrial cancer (with or without distant metastasis) (safety analysis set)

One patient with no reported clinical response was excluded from the analysis of PFS because their disease progression during the observation period could not be determined due to their withdrawal from the study.

Abbreviations: OS, overall survival. PFS, progression-free survival.





**Supplementary Figure 2.** Kaplan–Meier curves of (**A**) PFS and (**B**) OS in patients with endometrial cancer (with or without RT history) (safety analysis set)

One patient with no reported clinical response was excluded from the analysis of PFS because their disease progression during the observation period could not be determined due to their withdrawal from the study.

Abbreviations: OS, overall survival. PFS, progression-free survival. RT, radiation therapy.

**

**
